# Transcriptome sequencing of lncRNA, miRNA, mRNA and interaction network constructing in coronary heart disease

**DOI:** 10.1186/s12920-019-0570-z

**Published:** 2019-08-23

**Authors:** Jiangquan Liao, Jie Wang, Yongmei Liu, Jun Li, Lian Duan

**Affiliations:** 1 Department of Cardiology, Guang’anmen Hospital, China Academy of Chinese Medical Sciences, Beijing, China; 2 National Integrated Traditional and Western Medicine Center for Cardiovascular Disease, China-Japan Friendship Hospital, Beijing, China

**Keywords:** Transcriptome sequencing, Coronary heart disease, Long non-coding RNA, Micro RNA, Interaction network

## Abstract

**Background:**

Non-coding RNA has been shown to participate in numerous biological and pathological processes and has attracted increasing attention in recent years. Recent studies have demonstrated that long non-coding RNA and micro RNA can interact through various mechanisms to regulate mRNA. Yet the gene-gene interaction has not been investigated in coronary heart disease (CHD).

**Methods:**

High throughput sequencing were used to identify differentially expressed (DE) lncRNA, miRNA, and mRNA profiles between CHD and healthy control. Gene Oncology (GO), KEGG enrichment analysis were performed. Gene-gene interaction network were constructed and pivotal genes were screened out. Lentivirus-induced shRNA infection and qRT-PCR were performed to validated the gene-gene interactions.

**Results:**

A total of 62 lncRNAs, 332 miRNAs and 366 mRNAs were differentially expressed between CHD and healthy control. GO and KEGG analysis show that immune related molecular mechanisms and biological processes play a role in CHD. The gene-gene interaction network were constructed and visualized based on Pearson correlation coefficients and starBase database. 6 miRNAs in the network were significantly correlated to left ventricular ejection fraction, total choleterol and homocysteine. 2 lncRNAs (CTA-384D8.35 and CTB-114C7.4 (refseq entry LOC100128059)), 1 miRNA (miR-4497), and 1 mRNA (NR4A1) were the pivotal genes. Lentivirus-induced shRNA infection and qRT-PCR had validated the pivotal gene-gene interactions.

**Conclusions:**

These results have shown the potential of lncRNA, miRNA, and mRNA as clinical biomarkers and in elucidating pathological mechanisms of CHD from a transcriptomic perspective.

**Electronic supplementary material:**

The online version of this article (10.1186/s12920-019-0570-z) contains supplementary material, which is available to authorized users.

## Background

Cardiovascular diseases (CVD) have been recognized for years as the leading cause of death and long-term disability across the globe [1]. Despite all the concerns and effort devoted to preventing and treating CVD, the death rates have still increased significantly from 12.3 million in 1990 to 17.3 million in 2013, a 41% increase [2]. To better understand the aggressively increasing and uncontrolled CVD, especially ischemic heart disease, there is a need for continued research into the mechanisms at play.

Classic cardiovascular risk factors including hypertension, diabetes, and smoking can be used in disease stratification and prognosis prediction [3], molecular markers such as CRP and other serum inflammatory markers have gained increased attention in the exploration of the pathology [4], yet they are not sufficient to reveal the underlying molecular mechanisms of CVD.

With the advances in genomics and transcriptomics in recent decades, the discovery of the role of non-coding RNA’s (ncRNA), micro RNAs especially as well as long non-coding RNAs, in biological and pathological processes has added to the complexity of human genome [5–8], and has provided new approaches to study the molecular pathology of various diseases at a fundamental level.

Studies have shown the abnormal expression of micro RNA (miRNA) and long non-coding RNA (lncRNA) in CVD, including miR-1, miR-208, miR-499 in myocardial infarction [9–12], miR-126 in hyperlipidemia [13] and cardiovascular events [14], lncRNA ANRIL, KCNQ1OT1, MIAT, and MALAT1 in left ventricular dysfunction after myocardial infarction [15, 16], and lncRNA OTTHUMT00000387022 as novel biomarker for coronary heart diseases [17]. The extensive application of high throughput technologies including next-generation sequencing and microarray profiling has led to the screening and discovery of large numbers of differentially expressed (DE) ncRNAs that participate in the development of CVD, and has helped to gradually unravel the links between different types of ncRNAs and mRNA [18]. Exploration of non-coding RNAs, their targeting genes and their correlation with CVD is adding to our understanding of the pathology of CVD and providing new therapeutic targets for CVD.

In this study, we used RNA Sequencing (RNA-Seq) to identify DE lncRNA, miRNA, and mRNA in Coronary Heart disease (CHD) patients with unstable angina (UA), explore the co-expression network of lncRNA-miRNA-mRNA, and analyze the correlation between the clinical parameters and key genes in the network.

## Methods

### Participant recruitment and sample collection

This research complied with the Declaration of Helsinki and was approved by the Ethics Committee of Guang’anmen Hospital, China Academy of Chinese Medical Sciences. It has been registered in Chinese Clinical Trial Registry (ChiCTR) with the registration number: ChiCTR-BOC-16009239. All participants signed the informed consent voluntarily.

10 patients with UA (7 males) and 5 healthy control (4 males) patients were recruited in Guang’annmen Hospital, Beijing, China as the sequencing cohort. 30 UA (18 males) and 15 healthy control (9 males) patients were recruited in the same population as the validation cohort. All the subjects aged between 35 to 70 years old. The diagnosis of coronary heart disease (CAD) was confirmed in all UA patients by coronary angiography, showing at least one vessel lesion (> 50% narrowing of luminal diameter). All UA patients met the American College of Cardiology/American Heart Association (ACC/AHA) criteria for UA [19], and experienced ischemic chest pain within 48 h before the recruitment. The healthy controls were recruited from medical examination center of Guang’anmen Hospital, without chronic disease or infection in the last 2 weeks. General and clinical information including age, sex, history, complication(s), clinical parameters, and medication(s) were documented.

Patients who had received thrombolytic therapy in the previous month or with myocardial infarction, heart failure, valvular heart disease, dilated cardiomyopathy, malignant tumor, advanced liver disease, renal failure, autoimmune diseases, and other inflammatory diseases, and women who were pregnant or breast-feeding, were excluded from the study.

Whole blood samples (4–8 mL) were drawn from each participant. The blood samples were collected 48 h within the onset of chest pain in UA patients. Peripheral blood nuclear cells (PBNCs) were isolated using Red Blood Cell Lysis (TIANGEN, China) according to manufacturer’s instructions. Total RNA was obtained from PBNCs using RNAprep Pure Blood Kit (Tiangen Biotech, Beijing, China) following the manufacturer’s procedure. The RNA concentration and purity were checked by OD A_260_/A_280_ (> 1.8) and A_260_/A_230_ (> 1.6), and the yield and quality were accessed using Agilent 2100 Bioanalyzer (Agilent Technologies, Santa Clara, CA, USA), RIN number > 7.0.

### RNA library preparation, sequencing, and data processing

Library construction and sequencing were performed according to previously described methods [20, 21]. In brief, the whole transcriptome libraries were constructed using Ribo-Zero Magnetic Gold Kit (Human) (Illumina, San Diego, CA, USA) and NEBNext® Ultra™ RNA Library Prep Kit for Illumina (New England Biolabs) according to the manufacturer’s instructions. Libraries were tested for quality and quantified using the BioAnalyzer 2100 system and qPCR (Kapa Biosystems, Woburn, MA, USA). The resulting libraries were sequenced on a HiSeq 2500 instrument that generated paired-end reads of 100 nucleotides.

Raw sequencing reads were checked for potential sequencing issues and contaminants using FastQC. Adapter sequences, primers, number of fuzzy bases (Ns), and reads with quality scores below 30 were trimmed. Reads with a length < 60 bp after trimming were discarded. Clean reads were aligned to the human genome (GRCh38) using the TopHat 2.0 program [22], and the resulting alignment files were reconstructed with Cufflinks. The transcriptome of each sample was assembled separately using Cufflinks 2.0 program. All transcriptomes were pooled and merged to generate a final transcriptome using Cuffmerge. After the final transcriptome was produced, Cuffdiff was used to estimate the abundance of all transcripts based on the final transcriptome. We used the Potential Calculator (CPC) [23] to predict transcripts with or without coding potential. The read counts of each transcript were normalized to the length of the individual transcript and to the total mapped read counts in each sample, and expressed as fragments per kilobase of exon per million mapped reads (FPKM). Sequence reads mapped to different isoforms of individual genes were pooled together for subsequent comparative analyses. All the software mentioned above was set to the default settings.

### Small RNA library construction, sequencing, and data processing

Approximately 1 μg of total RNA was used to prepare small RNA library according to protocol of NEBNext® Small RNA Library Prep Set for Illumina. Single-end sequencing (50 bp) was performed on an Illumina Hiseq 2500 following the vendor’s recommended protocol.

Sequencing depth per sample pre and post QC filtering was 2X in RNA-Seq, and 1X in miRNA-Seq. Raw reads were checked for potential sequencing issues and contaminants using FastQC. Reads with a length < 10 nt and > 34 nt were discarded. The clean reads were aligned against the miRNA precursor/mature miRNA in miRBase20.0 (http://www.mirbase.org/) to identify known miRNAs. The unannotated sequences were mapped to the human genome to analyze their expression and distribution in the genome, and then used to predict potential novel miRNA candidates by Mireap (http://sourceforge.net/projects/mireap/). The read counts of each known miRNA were then normalized to the total counts of sequence reads mapped to the miRBase version 20.0 database and were presented as reads per million mapped reads (RPM).

### Sequencing data analyses and statistical methods

DE genes between CHD and healthy control were identified via Cuffdiff 2.0. |fold-change| ≥1, *P* value ≤0.05 and false discovery rate (FDR) ≤0.05 was considered statistically significant. Gene Ontology (GO) and KEGG pathway analyses were enriched through the Database for Annotation, Visualization, and Integrated Discovery (DAVID) 6.7 [24, 25]. Pearson correlation coefficient between DE lncRNAs, miRNAs and mRNAs were calculated respectively to construct the gene-gene interaction network. StarBase 2.0 (http://starbase.sysu.edu.cn/) was used to predict the interactions between DE genes, and the results were cross-referenced with the Pearson correlation analysis to enhance the reliability.

### Lentivirus induced shRNA infection in HUVECs

Human umbilical vein endothelial cells (HUVECs) were cultured in ECM supplemented with 10% fetal bovine serum (FBS). CTA-384D8.35, CTB-114C7.4 and miR-4497 lentivirus for overexpression studies were purchased from Hanbio Biotechnology (Shanghai, China). The lentivirus-packaged shRNAs (CTA-384D8.35, CTB-114C7.4, miR-4497) and empty vector of lncRNA and miRNA, and polyberne (5 μg/ml) were co-transfected into HUVECs (1.5 × 10^5^ cell per well) according to the manufacturer’s instructions. After 6 h infection, the supernatant was replaced with new ECM with 10% FBS. HUVECs were cultured for another 48 h and then harvested for quantitative real time RT-PCR (qRT-PCR) analysis.

### QRT-PCR analysis

QRT-PCR analysis was performed in the validation cohort and RNAi experiment samples. PBNCs extraction in the validation cohort and total RNAs isolation in PBNCs and HUVECs were performed as described above. RNAs were reverse-transcribed using Revert Aid First Strand cDNA Synthesis Kit (Thermo Scientific, USA) according to the manufacturer’s instructions. cDNA was analyzed by using 2X SYBR Green qPCR Mastermix (Roche Applied Science, USA). GAPDH was used as internal control for lncRNA and mRNA normalization, while U6 was used for miRNA normalization. Levels of gene expression were determined by standard curve analysis. The threshold cycle (CT) value was detected by 7900HT Fast Real-Time PCR system (Applied Biosystems, USA). Relative expression of target genes was calculated using the 2^-ΔΔCT^ method [26].

## Results

### RNA-Seq identifies lncRNAs, mRNAs and miRNAs are differentially expressed between CHD and healthy controls

Using lncRNA-mRNA RNA-Seq and miRNA-Seq, we have detected numerous transcripts in peripheral blood of CHD patients and healthy controls. Of these, 20,965 (91.35%) transcripts were known lncRNAs, whereas 17,152 (51.28%) transcripts were known mRNAs. As for miRNA-Seq, 8710 (50.38%) transcripts were known miRNAs. Applying the screening criterion established above via Cufflinks, a total of 62 lncRNAs, 366 mRNAs and 332 miRNAs were differentially expressed with fold change > 2.0, *P* < 0.05 and FDR < 0.05. Among them, 5 lncRNAs were upregulated and 57 were downregulated, 190 mRNAs were upregulated and 176 downregulated, 190 miRNAs were upregulated and 142 downregulated in the CHD group. The detailed information of DE RNAs were listed in Additional file 1: Table S1-S3 Additional file 2.

### DE lncRNAs, mRNAs and miRNAs distinctively classified the transcriptome profiles of CHD from healthy control

**Fig. 1:**
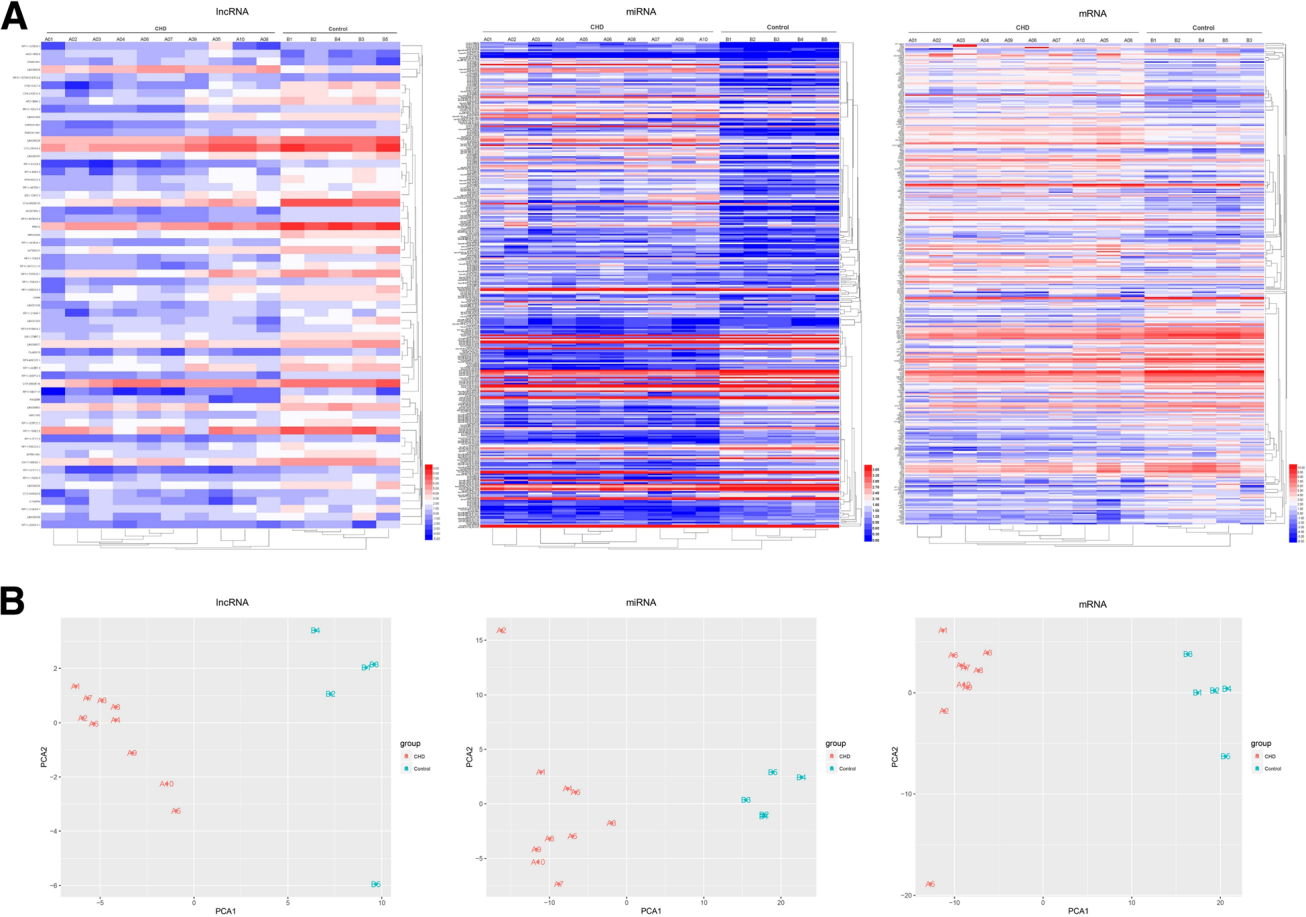
Heatmap and PCA analysis of DE lncRNA, miRNA and mRNA. The heatmap and PCA analysis showed that CHD and healthy control were categorized into 2 different groups by lncRNA, miRNA and mRNA

In order to understand the different characteristics between CHD and healthy control primarily, we conducted cluster analysis and Principle Component analysis (PCA) of the differentially expressed lncRNA, miRNA, and mRNA. Hierarchical clustering showed CHD and healthy control were well distinguished in lncRNA, miRNA, and mRNA, with all the subjects correctly classified (Fig. 1a). PCA also revealed distinct expression signatures of all 3 RNA types between CHD and healthy control (Fig. 1b). While previous studies have shown that miRNA and mRNA are differentially expressed in CHD [27–29], it is promising that lncRNA can also be used to discriminate between CHD and healthy control.

### Immune related molecular function and biological process of DE RNAs play a role in CHD

**Fig. 2:**
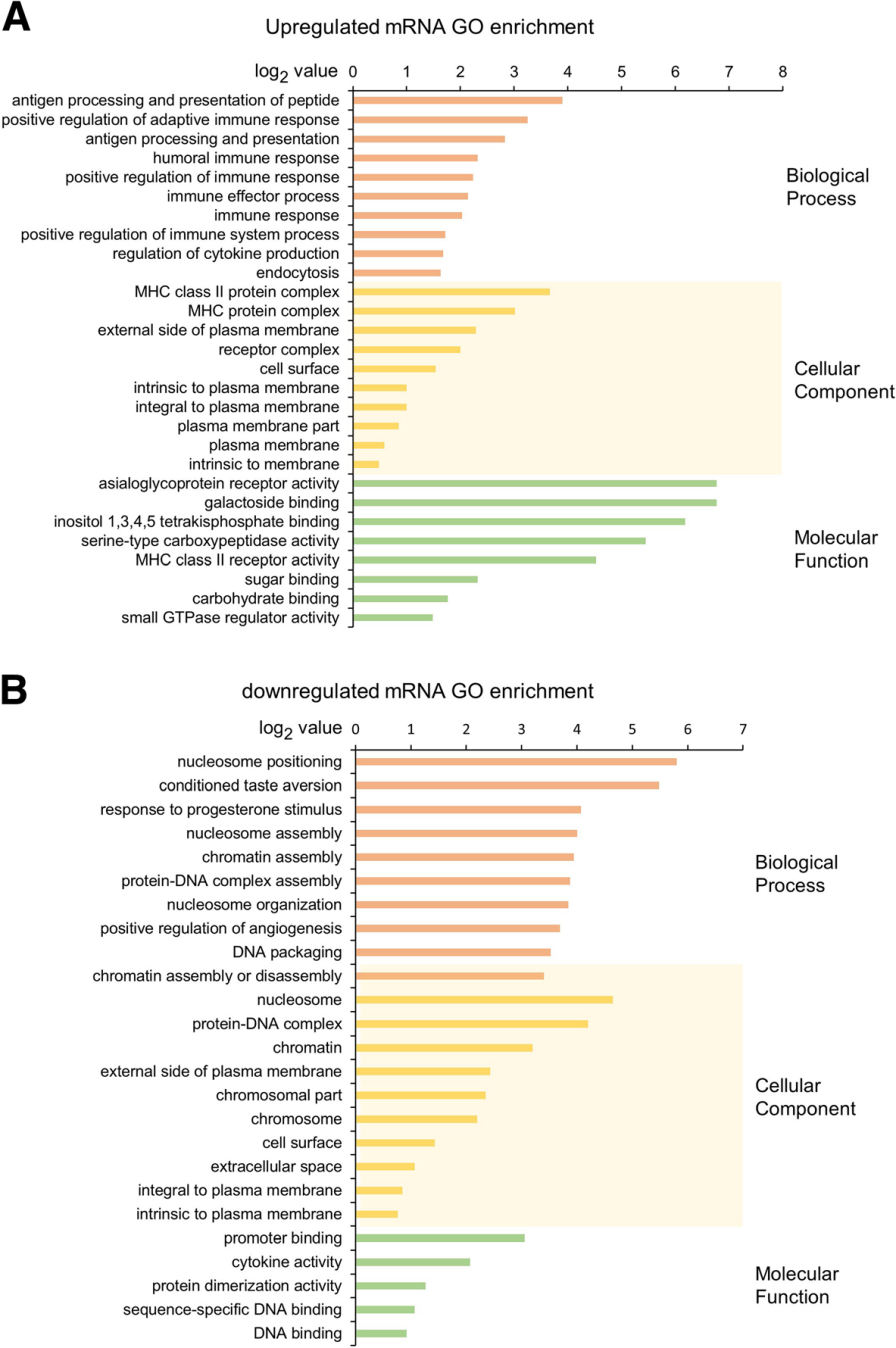
Top GO enrichment items of up- and down-regulated mRNAs. **a** and **b** showed the top GO enrichment items of up- and down-regulated DE mRNA respectively. The GO enrichment items were sorted by significance separately in Biological Process, Cellular Component and Molecular Function

The functions of lncRNA and miRNA are mainly exerted through regulation of expression of coding genes [18]. Hence, we analyzed the molecular functions of differentially expressed mRNAs using GO and KEGG databases, in order to reveal the role of related differentially expressed lncRNAs and miRNAs. GO enrichment and KEGG pathway enrichment analysis for upregulated and downregulated mRNAs were performed through DAVID 6.7. The top GO enrichment terms in upregulated mRNAs were *asialoglycoprotein receptor activity* and *galactoside binding*, ranked by enrichment score. The top enrichment term in downregulated mRNAs was *nucleosome positioning*. Apart from the basic cellular activities, immune related biological processes, such as *positive regulation of adaptive immune response*, *antigen processing and presentation*, *humoral immune response*, *positive regulation of immune response* and *immune effector process*, also showed up in GO enrichment analysis, especially in upregulated mRNAs (Fig. 2).

**Fig. 3:**
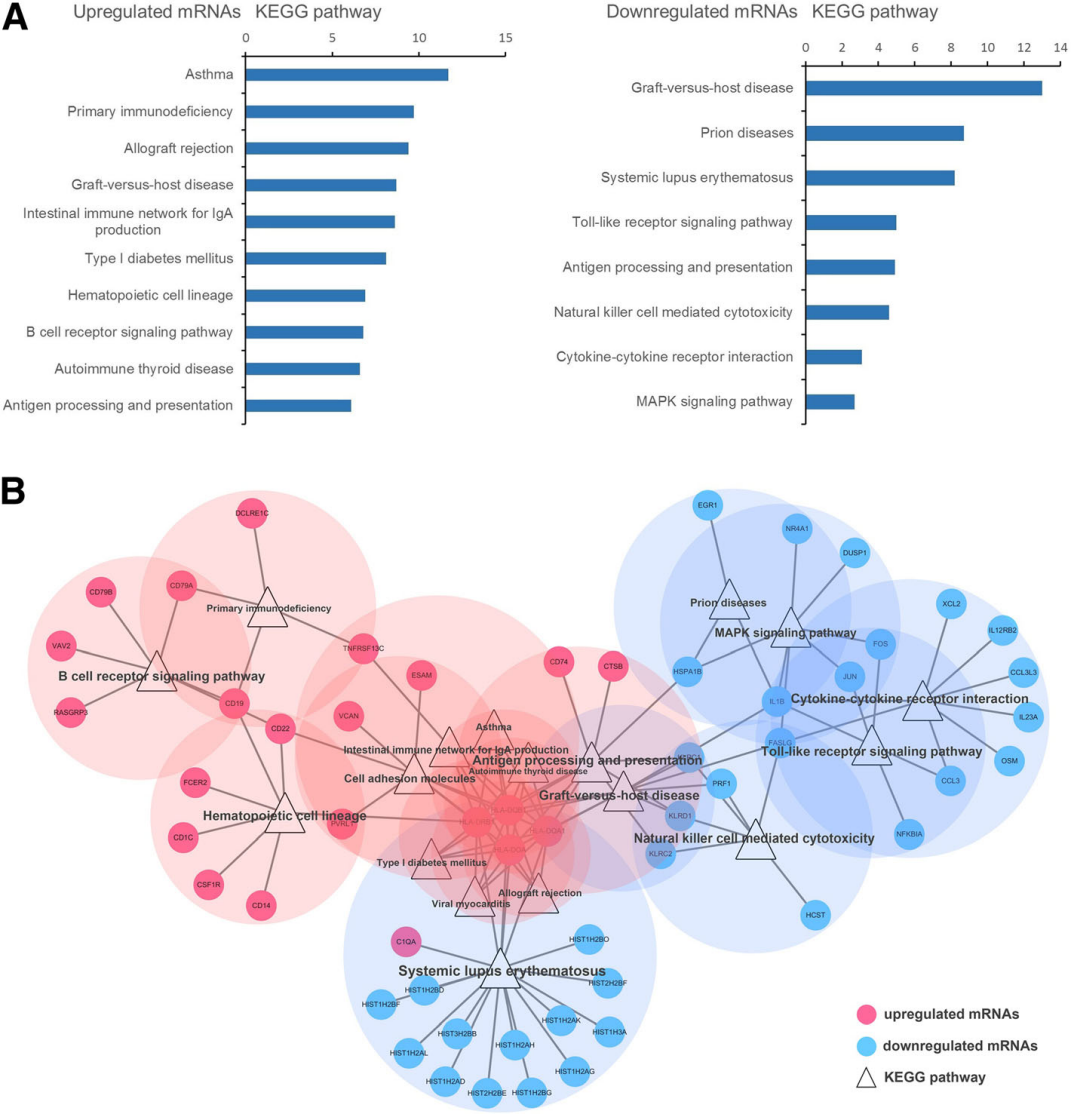
Top KEGG pathway enrichment of up- and down-regulated mRNAs. **a** listed the top KEGG enrichment items of up- and down-regulated DE mRNAs respectively sorted by significance. **b** mapped the DE mRNAs and KEGG pathways into 1 interaction network, and showed the overlap of different mRNAs in different pathways

The top KEGG pathway enrichments in upregulated mRNAs and downregulated mRNAs (Fig. 3a) included *Primary immunodeficiency*, *Allograft rejection*, *Natural killer cell mediated cytotoxicity*, *Cytokine-cytokine receptor interaction*, *Toll-like receptor signaling pathway* and *MAPK signaling pathway*. The DE mRNAs and enriched KEGG pathways are mapped in Fig. 3b. These results show that immune related molecular mechanisms and biological processes play a role in CHD.

### DE lncRNAs, miRNAs and mRNAs formed gene-gene networks by mutual interaction

It is evident that correlations exist between lncRNA, miRNA, and mRNA. To construct the gene-gene interaction network between differentially expressed lncRNA, miRNA, and mRNA, Pearson correlation coefficients and starBase were applied and cross-referenced to merge the gene-gene interactions into one consecutive network.

**Fig. 4:**
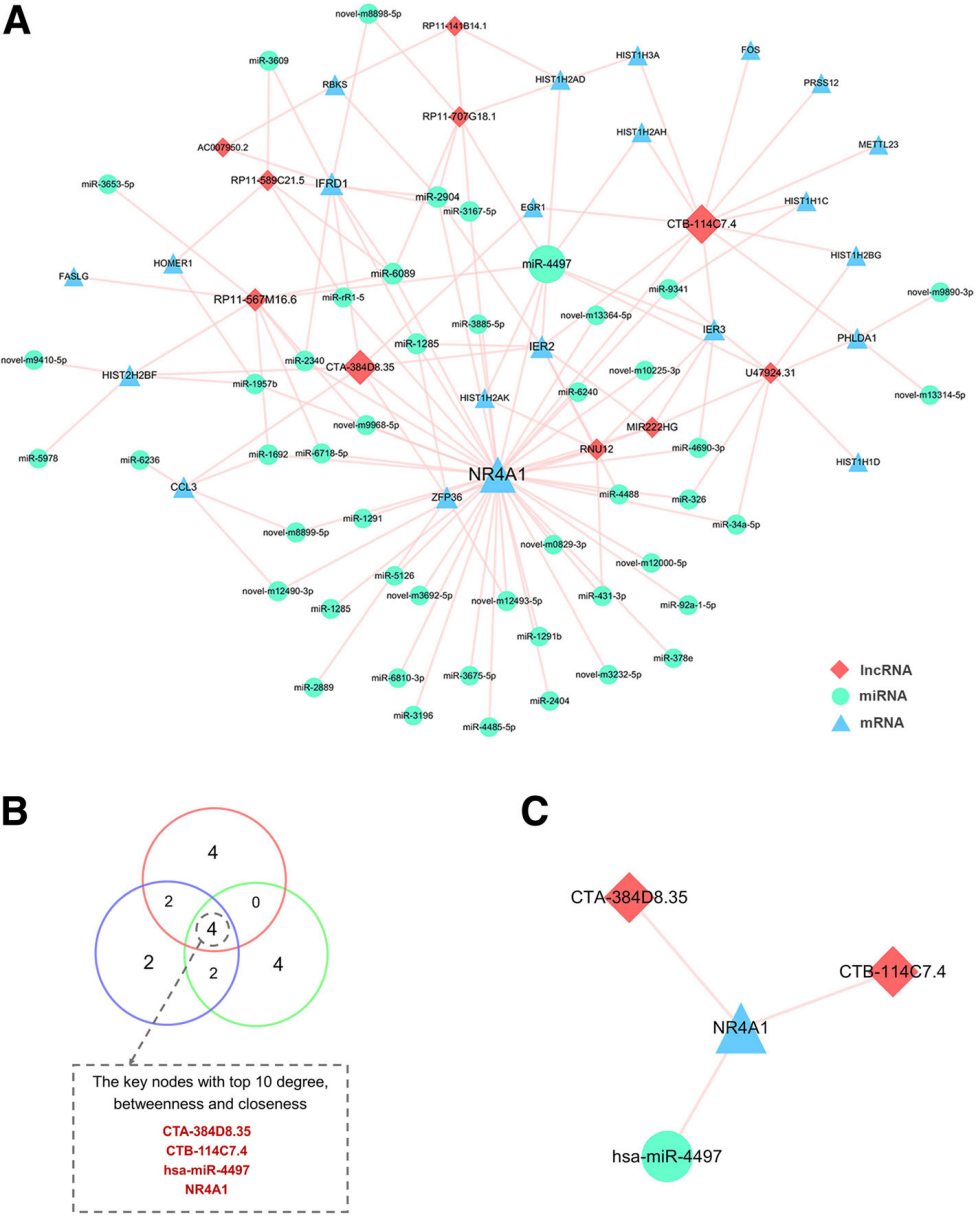
lncRNA-miRNA-mRNA interaction network of CHD related DE genes. DE lncRNAs, miRNAs and mRNAs with significance correlation were merged into gene-gene interaction network (**a**). **b** listed the 4 pivotal nodes in this interaction network based on the degree, betweenness centrality and closeness centrality. And **c** showed the interactions of this 4 DE genes

A total of 10 downregulated lncRNAs, 22 downregulated mRNAs and 51 upregulated miRNAs were included in this interaction network, containing 129 interactions. The interaction network was visualized using Cytoscape (Fig. 4a). To screen the key nodes in this interaction network, network tropical analysis was performed, and a Venn Diagram was drawn based on the degree, betweenness centrality and closeness centrality of the genes in the network. Four genes were found to be the pivotal nodes in the interaction network (Fig. 4b and c), which include 2 lncRNAs (CTA-384D8.35 and CTB-114C7.4 (refseq entry LOC100128059)), 1 miRNA (miR-4497), and 1 mRNA (NR4A1).

**Fig. 5:**
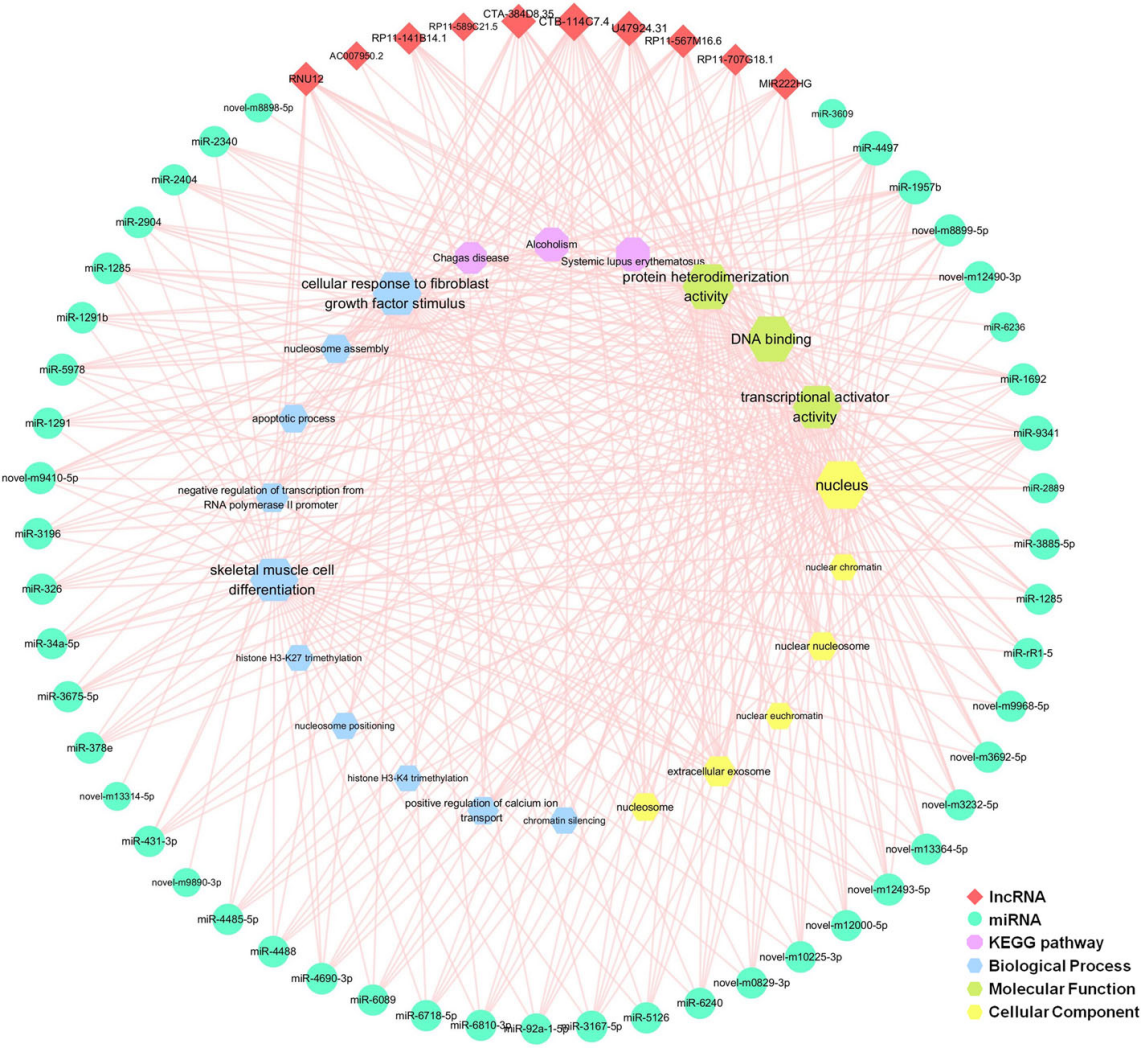
GO and KEGG pathway interaction with DE lncRNAs and miRNAs via mRNAs in gene-gene interaction network. This figure showed that DE lncRNAs and miRNAs in gene-gene interaction network may involve in these functional biological process via the DE mRNAs they interact with

The mRNAs in this network were uploaded to DAVID to perform GO and KEGG enrichment analysis. Twenty-two enrichment terms were obtained and linked to the targeted lncRNAs and miRNAs (Fig. 5). Histone and nucleosome related biological process formed a major part of the enrichment terms, such as *histone H3-K27 trimethylation* (enrichment score = 254.5), *histone H3-K4 trimethylation* (enrichment score = 101.8), *nucleosome positioning* (enrichment score = 190.8) and *nucleosome* (enrichment score = 61.7).

### Correlations exist between genes in interaction network and clinical parameters

It has been reported that certain DE genes can be correlated to disease related clinical parameters, some of which are more sensitive and respond quicker than conventional biomarkers [30–33]. The Pearson correlation coefficients between genes in interaction network and clinical parameters were calculated. Correlations with both *P* < 0.05 and |*r|* > 0.8 were considered correlated. 6 miRNAs were significantly correlated to 3 clinical parameters. Ejection Fraction (EF) was correlated to miR-326 (*r* = − 0.815, *P* = 0.0074), miR-4497 (*r* = − 0.878, *P* = 0.0019) and miR-92a-1-5p (*r* = − 0.808, *P* = 0.0085). Total Cholesterol (TC) was correlated to miR-3609 (*r* = − 0.814, *P* = 0.0075) and Homocysteine (Hys) was correlated to miR-2904 (*r* = − 0.834, *P* = 0.0052) and novel-m0829-3p (*r* = − 0.812, *P* = 0.0079).

### qRT-PCR validated the mutual interactions between pivotal genes in interaction network

**Fig. 6:**
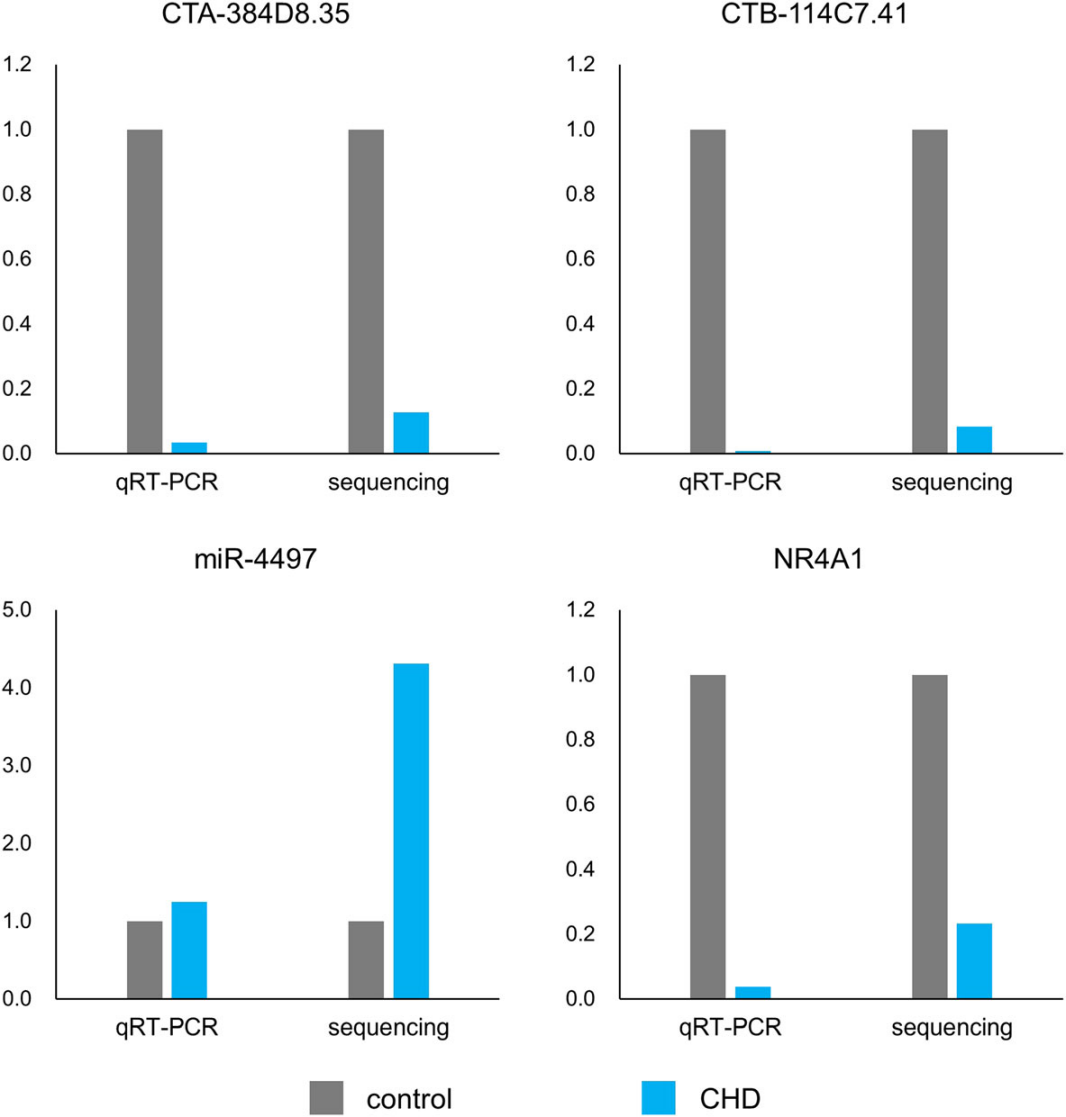
Validation of 4 pivotal genes by qRT-PCR. The expression of 4 pivotal genes in the interaction network were determined in a validation cohort. The results showed similar expression pattern in both validation cohort and sequencing cohort

The levels of pivotal genes including CTA-384D8.35, CTB-114C7.4, miR-4497, and NR4A1 were determined in a validation cohort by qRT-PCR. As shown in Fig. 6, all of these 4 genes were differentially expressed in CHD compared to control (*P* < 0.01 for CTA-384D8.35, CTB-114C7.4 and NR4A1; *P* < 0.05 for miR-4497). The expression pattern of these 4 genes in the validation cohort is similar to that in the sequencing cohort.

### Lentivirus-induced shRNA infection validated the gene-gene interactions

**Fig. 7:**
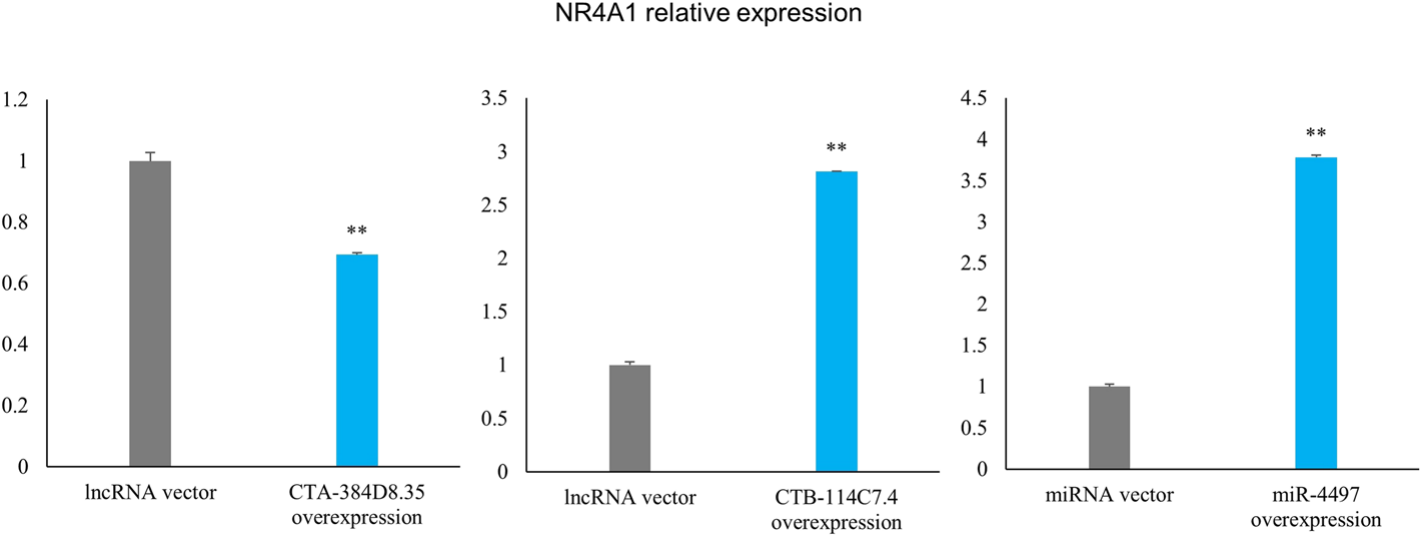
Validation of pivotal genes interaction via shRNA infection. NR4A1 expression level was altered after lentivirus-induced shRNA infection, confirmed the interaction between these 4 pivotal genes

The pivotal genes in the lncRNA-miRNA-mRNA interaction network, CTA-384D8.35, CTB-114C7.4, miR-4497 and NR4A1, formed a gene-gene interaction within themselves (Fig. 4c). To validate the gene-gene interaction, i.e., CTA-384D8.35-NR4A1, CTB-114C7.4-NR4A1 and miR-4497-NR4A1, we overexpressed CTA-384D8.35, CTB-114C7.4 and miR-4497 in Human Umbilical Vein Endothelial Cells (HUVECs). QRT-PCR analysis showed that the expression level of NR4A1 was upregulated in CTB-114C7.4 and miR-4497 overexpression compared to vector, while the expression level of NR4A1 was downregulated in CTA-384D8.35 overexpression (Fig. 7). These results validated the interaction within the lncRNA-miRNA-mRNA network.

## Discussion

Even though more than 80% of the human genome is transcribed, only 2% of the transcriptome is translated into proteins [34, 35]. Recent studies have shown the influential and vital role of ncRNAs in numerous physiological and pathological processes [36]. These ncRNAs include ribosomal RNA, transfer RNA, small nuclear RNA. Among them, miRNA and lncRNA have been studied for years and their involvement and interactions in various diseases are gradually being uncovered [30, 37–39]. It is widely reckoned that lncRNA, miRNA, and mRNA could interact with each other through different mechanisms [40].

However, a comprehensive analysis of lncRNA and miRNA in CHD has not been reported before. Here, we investigated the expression profiles of lncRNA, miRNA, and mRNA in CHD patients with UA using high throughput sequencing to explore the functions and interactions of lncRNA, miRNA, and mRNA.

A total of 5 lncRNAs, 190 miRNAs, 190 mRNA were found to be upregulated and 57 lncRNAs, 142 miRNAs, and 176 mRNAs were found to be downregulated significantly in CHD patients compared to that in healthy controls. These DE RNAs were distinctively classified by the disease groups in hierarchical clustering and PCA, suggesting a potential use for these ncRNAs in distinguishing CHD from healthy controls.

To determine the landscape of the molecular functions and associated pathways of DE RNAs, we performed GO and KEGG pathway analysis of DE mRNAs. Immune related terms were highly enriched in both GO and KEGG pathway analysis, including *positive regulation of adaptive immune response*, *antigen processing and presentation*, *humoral immune response*, *Primary immunodeficiency*, *Allograft rejection* and *Natural killer cell mediated cytotoxicity*. These results indicate that immune related molecular functions and biological processes participate in the pathogenesis of CHD.

It is well established that lncRNA can sponge miRNA via miRNA response elements (MREs) to interfere miRNA regulating its target mRNA, forming a competing endogenous RNA (ceRNA) regulatory network [41–43]. Hence, we performed Pearson correlation analysis among DE lncRNAs, miRNAs, and mRNAs, to identify the possible interactions between DE RNAs. We then cross-referenced starBase predictions of DE genes to enhance the reliability of interactions.

A lncRNA-miRNA-mRNA interaction network was constructed, including 10 lncRNAs, 51 miRNAs, 22 mRNAs, and 129 interactions (Fig. 4a). Most of the DE lncRNAs and miRNAs have not been studied yet, so we analyzed the functions of DE ncRNAs using the targeted mRNAs in the lncRNA-miRNA-mRNA network. GO enrichment analysis of DE mRNAs in ceRNA network showed that these DE genes were mainly involved in histone and nucleus related processes. Some of the DE miRNAs have been shown to be associated with CHD. Plasma miR-1291 was proved to be significantly overexpressed in acute myocardial infarction (AMI) patients in comparison with those without AMI [44]. MiR-326 inhibits Bcl-xL expression and induces apoptosis in platelets [45]. MiR-34a-5p indirectly regulates several hemostatic factors via decreasing the expressions of HNF4A and other mRNAs [46], and it was also detected as upregulated in atherosclerosis progression [47]. MiR-92a-1-5p has been identified as significantly increased in CHD patients, especially in those with UA compared to stable angina; it is also associated with high-density lipoprotein (HDL) [48]. Moreover, the inhibition of miR-92a-1-5p improves the re-endothelialization and prevents neointima formation after vascular injury of the femoral artery in mice [49].

In addition to these studies that showed cardiac related DE genes, the topological analysis of this ceRNA network showed that 2 lncRNAs (CTA-384D8.35 and CTB-114C7.4), 1 miRNA (miR-4497) and 1 mRNA (NR4A1) are the pivotal nodes as they ranked the highest in degree, betweenness and closeness centrality. Though CTA-384D8.35, CTB-114C7.4 and miR-4497 have not been studied yet, their functions could be interpreted by their target genes in the ceRNA network, including inflammatory responses (CCL3 [50], ZFP36 [51], IER3 [52]), apoptosis (EGR1) [53], atherosclerosis and myocardial ischemia (FOS) [54]. NR4A1 reportedly participates in myocardial diseases through various mechanisms, including cardiomyocyte apoptosis [55, 56], cardiac hypertrophy [57] and inflammation [58].

In vitro experiments validated the gene-gene interactions between CTA-384D8.35, CTB-114C7.4, miR-4497, and NR4A1, and showed the regulation of lncRNA and miRNA on mRNA, which may be the functional mechanisms of lncRNA, miRNA, and mRNA in CHD. It is worth noting that the RNAi experiment was performed with HUVECs. The interpretation of the results should be more objective and comprehensive. Future experiments with more ideal and vigorous design are needed.

We also analyzed the correlations between DE genes in ceRNA network and clinical parameters. 6 miRNAs were significantly correlated to EF, TC, and Hys. However, though the correlation coefficients were relatively high, these results need validations with a larger cohort. What’s more, lncRNAs and miRNAs are considered easier to degrade, and the production and secretion of them may change during the progression of myocardial ischemia. Hence a time course study is needed to explore the level of these DE genes in UA patients.

To conclude, we identified a profile of DE lncRNAs, miRNAs, and mRNAs that might be associated with the progression of CHD. These DE genes might function and interact mutually through ceRNA network. Our research suggests that specific lncRNAs, miRNAs, and mRNAs could be valuable as practical biomarkers for diagnosis, and as potential therapeutic targets of CHD.

## Additional files

Additional file 1 **Table S1** DE lncRNA detail information. **Table S2** DE miRNA detail information. **Table S3** DE mRNA detail information. (XLSX 38 kb)

Additional file 2 Sequence of pivotal ncRNAs in interaction network. (DOCX 15 kb)

## Data Availability

All data supporting the findings of this study are available within the manuscript except for the raw sequence data. Any data providing genotype information is considered to be personal property by the Chinese law, hence the submission to public achieves is prohibited. The raw sequence data can be acquired upon reasonable request from the authors (liaojiangquan@163.com) if approval could be granted from the Ethics Committee of Guang’anmen Hospital, China Academy of Chinese Medical Sciences.

## Abbreviation

AMI: acute myocardial infarction
ceRNA: competing endogenous RNA
CHD: Coronary Heart disease
CVD: cardiovascular diseases
DE: differentially expressed
EF: Ejection Fraction
GO: Gene Ontology
HUVECs: Human umbilical vein endothelial cells
lncRNA: long non-coding RNA
miRNA: micro RNA
ncRNA: non-coding RNA
PBNCs: Peripheral blood nuclear cells
TC: Total Cholesterol
UA: unstable angina
