# Supplementary material for: Transcriptome sequencing of lncRNA, miRNA, mRNA and interaction network constructing in coronary heart disease
Source: BMC Med Genomics. 2019 Aug 23;12:124. doi: 10.1186/s12920-019-0570-z (PMC6708182; doi:10.1186/s12920-019-0570-z)
Supplement: Supplementary file 2 — Sequence of pivotal ncRNAs in interaction network. (DOCX 15 kb) [file 12920_2019_570_MOESM2_ESM.docx]

**Sequence of pivotal ncRNAs in interaction network**

lncRNA:

1. CTA-384D8.35 (locus: 22:50542304-50543011, length: 602 bp):

TTCTTAGGCGTCCTCATCCCTTTTCACCTCGGAGCTCAGCGTCTTCCTCAGCAGCACTTCCATGTCATCTGCCCCGTGAAATCAGCCTAACGCCGTTTCTCAATGACGTGGATCGCCCTAGGCCACCGCAACCTTCCGGAAGCTCTCTCAGCTCAGTTCCCATTCTCCCACCATCTCTTGGTTCTCCTTCTACCTCACCGGTTGCTAGTCCTCCGCTTCGCAGCTGAAAATGTGCCCGGGGCCTACTGTGGGCCTAGCCAGGCCTGCTTACGCAGTGCAGTTTCCCATGAATGATGCCCAGTCATTATCACATAACCTGTGGCAAGCCAGCAAGATGGCCCTGGTGACAGCAAAAGAAACTGCACTAGGACCTGAATGTAGATCTCAGTCATGTTCCTTACTAACAGCACGTTTTGCAACCATGCGTTAAAGAAACATCTGACTCACAACAAAATTTTAAAGGGTTTATTTGAGTGAAAAGCAATTTATGAATTGGGGAACACCTGACTGAAAGAGCGTTAGTATTCCAGAGACAAAACATCAAGTGCAAGTTTTTATTGGGAAAATGTAGAAGCACAATAAAGAAATTATTTGATTGGTTA

2. CTB-114C7.4 (locus: 5:170308700-170312716, length: 2182 bp)

ATGTGTTCTGCTTTCCCAGGGTCCATGGCAGGAGGGCTGCAGCGGCCTCATTTATTCATTTGTGCCAGGCACCCTGTTAATCATGGAGATACCACGCTGACCTGCCTTCAAGGAGACCATATTCTAGTAGGAGAGCTGAGCAGCGAGATGGCCATGTGTAGATGGTGCTATATGAAAGATCACCCTGATGTAGAAGCCAGGTGGGGCATCTTGGCTTCCTGCTCCTTTTCTTTCATATGACACCGAAAAGTCTCTGGATATTGCAGTTGGGCCAGACCTGCAGAAAAAGAGAACATTCAGTTAAACAGAAGAACATATTCTGGAGAAAATGGGGAAGAGAGTGAAGACACAGCTGGTTCAAGGGAAAAATGCCAACTGAGCACAGAGAGAAAAATGCCTGCTGAGAGCTCCACAAATAAAGAAAAAACTTACATAAAGTGGTGAGAAGCACAGGAGAGGACAGGGCCGGGGCTGAGAGGCTGAAGTTCCAGGACCGGTTCTGCATTTGCTGCTGTGTGACATGGGGCCAGTGACTTCCCATCTCTGGGCTTCAGTTTTCTCAATTATCATCTATTCTCCTGCTTTCTCTATAACATTCATTTATTGATTCCTTGATTCAAGAATATTTCTTAAGCACCCAGTTTGTGCCAGGTACAGCTGTCAGTGCTAGTGATTCAGCAATGAACAAAGTGGACAAAAAGCACATCCTTATGTGAGCCCTCATGCATGCAGCAAACACAATCAGTGAAATATGGTGCAGATTCATTTGTAATAAGGAGAAAACAGAAAGCACAGAAGGGGTCAGGCAGTCTAATCTGGAAGGGTGACCTCTGAGTCAAGACATAAGGAGGTGAGGGAGTGAGCCAGGAGGACATCTGGGCAAAGCCTGCTCCAGGCAGAGGGGACAGCCAGTGTGAGAGCTGCCCTGAATGCAAAGCCTACCCACTGCCCCTTCTTTTGGTGTCTCCACTGGCCTGCTGGCTGCAGCAGCCTTCTATCAGACCTCCCAGCCTCCATCTCCTCCCCTGGGGTCTTCTAGCCCCACATGGCCAGCCAGGGTGACTCTAAAATATAAGTCAGACCCCAACACGTCCCTGCTCAAAACCCACCAGGTGCTCCCACCACATTCAATGCAGTGCAAGTCCTCACCATGGTCCTCTAGCATCTCTCCAAGCCCATCTGCCTCCTCTCTCCCTCACTTGCTCCTTTCCAGCTACACTGGCCTCTGCTGTACCTGGGACATGCCAAGCAAGACCCAGCCTTGGGGTCTTTGCTCACGGCCTCTGCCTGGATGTTCTTCCCCCAGTTATCCACAGGTCTGGCTTCCTGACTTTATTCAGAGGCCACCCTCTCAAGAAGACACCCTTGACTGTTTCGTCTAAGCAAGACCCTGTCACATCCAACCACTCGCCTCGCTTTATTTTCCCTTACAGCATTTATCATGACCTCCCATCATTTTATATATTTATAATAGTAGATATGATCAATGGATCAGATGTTTATTATGTGATGGACACTGTTCTAAACATCCTAGAATTAATTCATCTTCTCCCAAACTCCTTGAGATTGGTATTGCCATTGACCACATTTTATATATCAATAACCCAAGATAGAGAGAGGTTAAGAAATTTGCTCAAGGTCTCCCATCTGGTGAGTGTCAGATCAGGATTCAAACCCTGGAGCTTCCATTATGAGCCACAACACTCTAGACCAGGGCTGCCCAGTAGAACTTTGCAATGATGAAAATGTTCTGTGTCTGCACTGCCCGAAGTGACTGCCACTGGATATGTGTAAGTGTTGAGCATTTGAAATATGTGTGACCAAGGAACTGAATTTTGAATTTTATTGCATTTCCATGACTTTAGATTTAAATAGCCAGGCTAGTGGCTACCAAATTGTACATAGAGCCCTCGCACCTCTTATTTGCTCCTTTGTTAATTTCTGTCTGCCACATAAGGATGAAACTTGCCTATGCATTAGTTTCCTCATTTTACAGTAAGGGAAACACCCCTGGTACAAAGTAAATACTCAATGCTCATTAATCACCATCATCATCACCATTATTATAACATTATCATTATTAGTCTCCATGAGATCGATGACTTTATGTTTGTTCACTCCTGAATCCCATTACATTCTTTAGCACATAGCAGGTGTTCAATAAATATATTATGAATGAATG

miRNA:

miR-4497 (region: R1-16L17, length: 34 bp):

CGGTGCGCCGCGACCGGCTCCGGGACGGCTGGGA
